# Duration invariance and intensity dependence of the human circadian system phase shifting response to brief light flashes

**DOI:** 10.1098/rspb.2021.1943

**Published:** 2022-03-09

**Authors:** Daniel S. Joyce, Manuel Spitschan, Jamie M. Zeitzer

**Affiliations:** ^1^ Department of Psychiatry and Behavioral Sciences, Stanford University, Stanford, CA, USA; ^2^ Mental Illness Research Education and Clinical Center, VA Palo Alto Health Care System, Palo Alto, CA, USA; ^3^ Department of Psychology, University of Nevada Reno, Reno, NV, USA; ^4^ Translational Sensory and Circadian Neuroscience, Max Planck Institute for Biological Cybernetics, Tübingen, Germany; ^5^ TUM Department of Sport and Health Sciences (TUM SG), Technical University of Munich, Munich, Germany

**Keywords:** alertness, circadian, light, melatonin, sleep, flash

## Abstract

The melanopsin-containing intrinsically photosensitive retinal ganglion cells (ipRGCs) are characterized by a delayed off-time following the cessation of light stimulation. Here, we exploited this unusual physiologic property to characterize the exquisite sensitivity of the human circadian system to flashed light. In a 34 h in-laboratory between-subjects design, we examined phase shifting in response to variable-intensity (3–9500 photopic lux) flashes at fixed duration (2 ms; *n* = 28 participants) and variable-duration (10 µs–10 s) flashes at fixed intensity (2000 photopic lux; *n* = 31 participants). Acute melatonin suppression, objective alertness and subjective sleepiness during the flash sequence were also assessed. We find a dose–response relationship between flash intensity and circadian phase shift, with an indication of a possible threshold-like behaviour. We find a slight parametric relationship between flash duration and circadian phase shift. Consistent with prior studies, we observe no dose–response relationship to either flash intensity or duration and the acute impact of light on melatonin suppression, objective alertness or subjective sleepiness. Our findings are consistent with circadian responses to a sequence of flashes being mediated by rod or cone photoreceptors via ipRGC integration.

## 1. Introduction

The human circadian system is exquisitely sensitive to light. Light exposure (LE) in the evening and night can acutely suppress the production of melatonin [1–6], shift the phase of the circadian clock [5,7–10] and modulate alertness and vigilance [11–13]. These effects are mediated by the retinal photoreceptors, with a major role played by a subset (less than 3%) of the retinal ganglion cells that express the short-wavelength-sensitive photopigment melanopsin, rendering them intrinsically photosensitive [14]. These intrinsically photosensitive retinal ganglion cells (ipRGCs) also receive cone and rod input [15], which contribute to a complex signal driving the circadian system. The exact effect of a given light on the circadian system depends on its intensity, spectral distribution, duration and circadian phase of administration [16–18]. While experimental durations of LE are typically on the order of hours, it has been shown that sequences of 2 ms flashes of bright light (approx. 1700 lux) can induce phase shifts in humans that are substantially larger than continuous light of the same illuminance [19].

**Figure 1.**
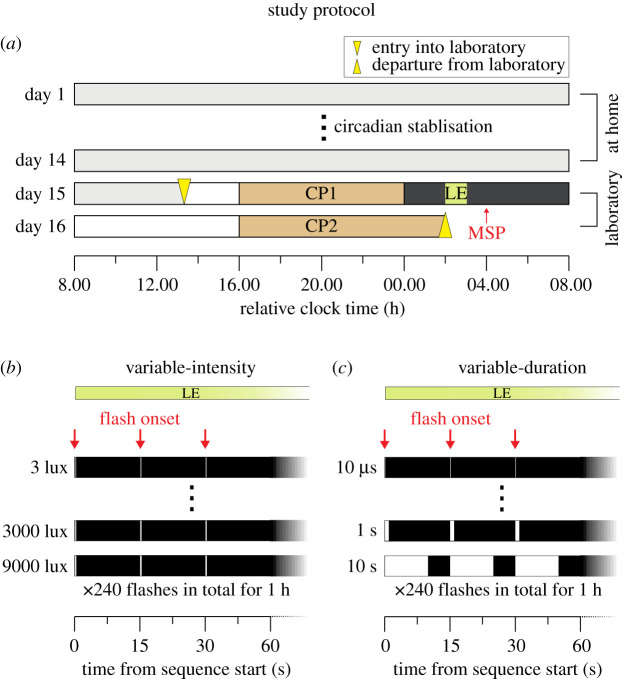
Circadian phase shifting protocol. (*a*) Schematic diagram of 16-day study protocol. During CP1 and CP2, saliva samples were collected and behavioural assessments (objective alertness, subjective sleepiness) were performed. MSP, mid-sleep point; LE, light exposure; CP, constant posture. (*b*) Schematic diagram of flash sequence over the 1 h light exposure phase. Flashes at fixed duration (2 ms) were separated by 15 s onset-to-onset and varied between 3 and 9500 lux. (*c*) Schematic diagram of flash sequence over the 1 h light exposure phase. Flashes at fixed intensity (approx. 2000 lux) were separated by 15 s onset-to-onset and varied between 10 µs and 10 s. Spectral properties of the stimulus were invariant of flash intensity and duration (electronic supplementary material, figure S2), and for variable-duration stimuli the total time-averaged radiance delivered was as expected, scaling linear with duration. (Online version in colour.)

Here, we systematically investigated the temporal integration properties of the human circadian system in a 34 h in-laboratory between-subjects design (figure 1). During the biological night, we exposed healthy observers (*n* = 28) to a 60 min sequence of short-duration white light flashes that varied in flash intensity over 4.5 orders of magnitude (3, 30, 95, 300, 950, 3000 or 9500 photopic lux) at fixed duration (2 ms) and measured the consequent impacts on circadian phase, melatonin suppression and alertness. Additionally, we examined how short of a flash the human circadian system could respond to by examining sequences of short-duration light flashes spanning six orders of magnitude (10 µs, 100 µs, 1 ms, 10 ms, 100 ms, 1 s, 10 s) at fixed intensity (2000 lux). Stimuli were presented using eye masks, illuminating the retina with a homogeneous full-field of light. Our results provide mechanistic insight into how the human circadian system integrates environmental information of ambient illumination.

## 2. Results

### (a) Circadian phase shifts to 2 ms flashes are intensity-dependent and robust

**Figure 2.**
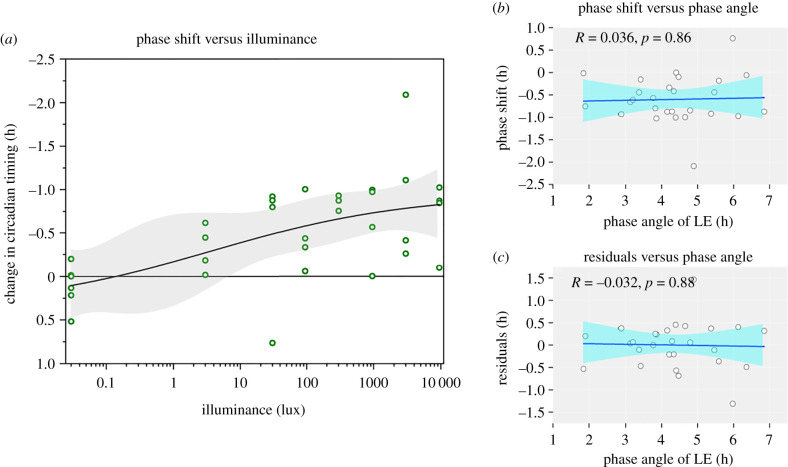
Flashes of light shift circadian phase in an illuminance-dependent manner. Dose–response curve for circadian phase shifts across four orders of magnitude of photopic illuminance (2 ms flashes delivered every 15 s for 60 min) measured in an in-laboratory between-subjects design (*n* = 27). Individual, per-subject data points are shown as white circles. A four-parameter dose–response curve (black line) with 95% CI (shaded grey) fitted to these data is overlaid. There is no relationship between the timing of light exposure and the phase shift (*b*) or the residuals in the linear model (*c*). (Online version in colour.)

We first examined our variable-intensity dataset for a dose–response relationship between the intensity of a white, broad-spectrum flash (CIE 1931xy chromaticity: [0.4092, 0.3969], correlated colour temperature (CCT): 3466 K; melanopic efficacy of luminous radiation (ELR): 0.72) measured in photopic illuminance, and the shift of the circadian clock measured as the difference in dim-light melatonin onset (DLMO) on subsequent evenings. Flash stimuli were delivered during the biological night as full-field homogeneous stimuli using light masks and carefully calibrated in the spectrum and temporal properties (electronic supplementary material, figures S1 and S2). The phase angle of LE (i.e. the time between DLMO and the onset of the light stimulus) was similar among participants, with the onset of light being 4.35 ± 1.25 h after DLMO. While there was variability in the exact phase of application of the light stimulus, this variability did not appear to impact the magnitude of the phase shifts (figure 2*b*,*c*). One participant had a mistimed light stimulus as the melatonin onset occurred during sleep (Grubbs's test, *p* = 0.05 threshold); this participant's data were excluded from all further analyses. A linear function provided a reasonable fit to the data (adj *r*² = 0.32, *p* < 0.001), with a slope estimate of −0.19 ± 0.11 h/lux. We also fitted a four-parameter dose–response function to the data, which also generated a reasonable fit (adj *r*² = 0.28, *p* < 0.001). Parameter estimates from this function indicate a maximum shift is −0.86 ± 0.68 h, a minimum shift (due to protocol) of 0.37 ± 1.8 h, a power term of the slope (indicating the steepness of the *s*-curve) of 0.30 ± 0.76 and a sensitivity term (intensity at which half of the maximum shift is observed) of 3.9 ± 27 lux (figure 2*a*; note the logarithmic scale).

### (b) Circadian phase shifts to 2000 lux stimuli are duration-independent when delivered as a sequence of micro- or millisecond flashes

**Figure 3.**
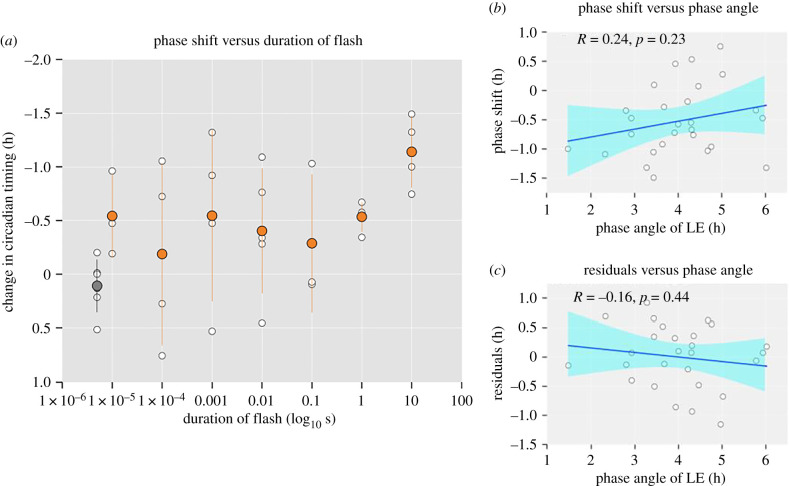
Dose–response relationship for circadian phase shifts across six orders of magnitude of flash duration (2000 lux flashes delivered every 15 s for 60 min) measured in an in-laboratory between-subjects design (*n* = 27). Individual, per-subject data points are shown as white circles, group mean ± s.d. estimates are shown as filled orange circles. Note, the control condition (filled dark grey circle) is arbitrarily plotted at 5 × 10^−5^ for visualization purposes only—there is no light in this condition so the duration would be 0. These data were unable to be fitted with a dose–response. There is no relationship between the timing of light exposure and the phase shift (*b*) or the residuals in the linear model (*c*). (Online version in colour.)

We next examined the effect of a regime of 240 moderately bright flashes (approx. 2000 lux) of the same white, broad-spectrum light spectrum but of varying duration (individual flash lengths of 10 µs, 100 µs, 1 ms, 10 ms, 100 ms, 1 s, 10 s; 15 s duration onset-to-onset). The phase angle of LE (i.e. the time between DLMO and the onset of the light stimulus) was similar among participants, with the onset of light being 4.01 ± 1.06 h after DLMO. While there was variability in the exact phase of application of the light stimulus, this variability did not appear to impact the magnitude of the phase shifts (figure 3*b*,*c*). One participant had a mistimed light stimulus as the melatonin onset occurred during sleep (Grubbs's test, *p* = 0.05 threshold); this participant's data were excluded from all further analyses. A linear function provided a moderate fit to the data (adj *r*² = 0.17, *p* < 0.05), with a slope estimate of −0.074 ± 0.030 h s^−1^. When data from individuals exposed to a sequence of 10 s flashes were removed, there was no longer a significant linear fit (*p* = 0.38, adj *r*² = 0.008). We attempted to fit a four-parameter dose–response function to the data, with the a-term (protocol-based shift) set at 0.11, but the fit failed to converge. Across different flash durations, we find phase delays in circadian timing on the order of 0.52 ± 0.59 h (figure 3*a*). This is different from the protocol control condition in which advances of 0.11 ± 0.25 h are observed (*t*-test, *p*'s < 0.05 with or without 10 s data). While there was a linear trend for increasing flash duration when including the 10 s data, direct comparison of the different duration of flashes, spanning six orders of magnitude (1 : 1 000 000), indicated no difference in the response to the different durations (*F*_1,25_ = 0.0018, *p* = 0.97).

### (c) No evidence for illuminance-graded acute effects of a sequence of ultra-brief light flashes on acute melatonin suppression, objective alertness or subjective sleepiness

**Figure 4.**
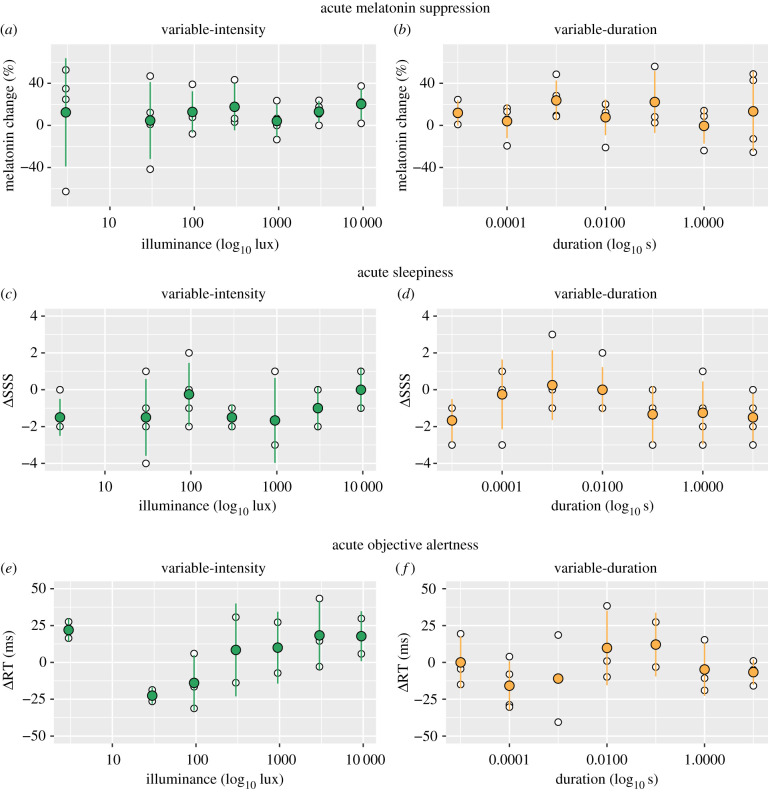
Flashes of light do not affect acute non-visual effects of light reliably. (*a*,*c*,*e*) Measurements of acute melatonin suppression, acute sleepiness and acute objective alertness in the variable-intensity protocol. (*b*,*d*,*f*) Measurements of acute melatonin suppression, acute sleepiness and acute objective alertness in the variable-duration protocol. Individual, per-subject data points are shown as white circles, mean ± s.d. estimates are shown as green (variable-intensity; left column) and orange (variable-duration; right column) circles. (Online version in colour.)

We examined whether light flashes elicit acute changes in melatonin production, objective alertness (median reaction time measured using an auditory psychomotor vigilance test, PVT [20,21]), and subjective sleepiness (Stanford Sleepiness Scale, SSS [22]). We compared these endpoints just before administration of the light stimulus to the end of the hour of light administration (figure 4). We find no effect of flash illuminance on acute melatonin suppression (*F*_1,25_ = 0.17, *p* = 0.68), objective alertness (*F*_1,24_ = 0.00084, *p* = 0.98) or subjective sleepiness (*F*_1,25_ = 0.86, *p* = 0.36). We find a significant decrease in subjective sleepiness independent of flash illuminance (−1.15 ± 1.67; *t* = −2.37, *p* = 0.0261), consistent with the non-specific effects of being awakened at night. Likewise, we find no effect of flash duration on melatonin suppression (*F*_1,25_ = 0.023, *p* = 0.88), objective alertness (*F*_1,25_ = 0.11, *p* = 0.75) or subjective sleepiness (*F*_1,25_ = 0.85, *p* = 0.37). As with the variable-duration results, we find a significant decrease in subjective sleepiness independent of flash duration (−0.741 ± 1.56; *t* = −2.40, *p* = 0.0244).

## 3. Discussion

Previous studies involving exposure to single episodes of continuous light indicate that the circadian system does not act like a simple photon counter, as the combination of duration and intensity of LE are not additive [23]. In our current study, we demonstrate that in response to sequences of brief flashes of light, the human circadian phase-shifting system too does not simply act as a photon counter and does not integrate over intensity and duration equally. Long-duration (6.5 h) continuous LE presented during the early biological night [5] demonstrates a clear dose–response relationship between light intensity and magnitude of the phase delay. The flash data also demonstrate a dose–response relationship, albeit with a steeper step function, with a sensitivity of approximately 4 lux. In response to a sequence of light flashes, the human circadian system is at least a log unit more sensitive than it is in response to continuous light. In parallel, at the intensity of light tested, changes in the duration of bright flashes of 1 s or shorter seem to have little impact on the circadian system, as we observe invariant responses to a 1 : 100 000 difference in flash durations. These data are consistent with light flashes being mediated through mechanisms, either at the photoreceptor or circuit level, that differ from those mediating responses to continuous light.

Much of the impact of continuous light on circadian function is thought to be mediated through the intrinsic (melanopsin) rather than extrinsic (rod/cone) photoreceptive circuits, especially for short-wavelength monochromatic light [24]. Our data, however, suggest a possible mechanism for temporal integration through a relative increase in the outer retinal rod and/or cone contributions to circadian photoreception via the extrinsic ipRGC pathway. This is consistent with recent data from mice in which melanopsin has been shown to be unnecessary for responses to flashes of light [25]. In rodents, outer retinal inputs to the suprachiasmatic nucleus (SCN) are both measurable [26] and functionally significant in driving photoentrainment, as melanopsin-knockout mice can stably entrain to an LD cycle but exhibit attenuated circadian phase shifting to a single pulse of monochromatic blue light [27]. A subset of primate ipRGCs, the M1 subtype, receive excitatory input from the L and M cones, and inhibitory input from the S cones [15] and extrinsic input from human rods and cones to ipRGCs has been described in an *in vitro* preparation [28]. Indeed, the extrinsic contribution of rods and cones to ipRGC signalling appears exceed additive excitation [28]. However, while there is converging evidence for connectivity of outer retinal inputs into ipRGCs, the specific role rods and cones play in circadian entrainment remains unclear. Recent data from mice has indicated that cones may be specifically important for conveying information about rapidly changing light levels [29], the exact circumstance being tested with a sequence of light flashes. Cone signalling rapidly adapts under continuous light paradigms that greatly reduces its ability to signal for non-image-forming functions [30,31]. For the circadian system at least, rod contributions may be preserved even at photopic irradiances and drive photoentrainment [30,32], and it has been hypothesized that cone or rod inputs may optimally detect dynamic changes in sunlight intensity and spectrum that occur during dawn or dusk [25].

With our flashed-light paradigm, the brief flashes in combination with the relatively long 15 s inter-stimulus intervals (ISIs) may allow rods to at least partially regenerate in the darkness [33,34] while the ipRGC continue to fire as if continuously stimulated [26]. Because rods make up the large majority of photoreceptors in the human retina, this contribution might not be insignificant. Future studies of *in vivo* ipRGC circuit electrophysiology, coupled with human studies using monochromatic light or photoreceptor-selective stimulation paradigms [35,36] (rather than polychromatic, photoreceptor non-selective white light as used in this study), could clarify photoreceptor contributions to this process. It should be noted that there is a possibility that the responses to the sequences of 10 s ‘flashes' of light may not be appropriate to compare to those of the sub-second flash sequences and may be more comparable to responses following intermittent light protocols [18,37] or protocols that use exposure to single, short pulses of light [16]. These 10 s flash sequences had only 5 s of darkness between each LE, which could elicit a resensitization process that is fundamentally different than the one occurring between sub-second light flashes, which has threefold more time in the darkness between each flash. Indeed, when the 10 s data were removed from analyses, we no longer observed a significant relationship between the duration of the light flashes and the induced circadian phase shift.

The ability of humans to consciously perceive light spans a very wide range of light intensities, from the sensitivity to single photons by the retinal rods (e.g. [38]) to encoding fine spatial detail, colour and motion during daytime light levels. At detection threshold, the intensity and duration of a flash can be traded off against one another below a certain flash critical duration, leading to the same conscious perceptual performance if the product between the intensity and the duration is the same. For conscious perception, this temporal integration does not hold for flash durations over approximately 100 ms [39], thought it persists greater than 1 s for pupil responses [40]. For shifting the circadian clock, however, it appears as though the mechanisms integrating light information are different and are likely to be nonlinear, as shown in the results here.

We note that the variability of individual responses to flashed light is higher than that of continuous light, but commensurate with other studies of flashed lights in humans [19,41] and flies [42], and with the recently identified large individual differences in circadian sensitivities to evening LE [43]. In comparison to continuous light paradigms, flashed light paradigms may be more susceptible to probabilistic photon catch over the short stimulus windows, which may explain the phase advances or near-zero phase shifts seen at low durations (sub-millisecond) in the variable-duration experiment as participants would be more likely to experience a slight non-specific advance that we observe in the control arm of this protocol. The total stimulus duration over 1 h is reduced 7500-fold compared to an hour of continuous light or 48 750-fold compared to 6.5 h of continuous light. In principle, differences in pupil size (which change the retinal illuminance) can alter the input to non-photic processes (at the same nominal corneal illuminance) [44]; however, the pupil only constricts to light onset with a delay of approximately 200 ms [45], making our variable-intensity flashes at 2 ms robust to such an effect. While there may be a cumulative effect of our sequence of flashes on long-term steady-state pupil size, the pupil will nonetheless reach significant redilation after our long 15 s ISI. While the variable-duration measurements at 1 and 10 s may be more susceptible to any such effect, we do not see a strong phase delay at the examined illuminance (2000 lux).

Flashed light confers advantages over continuous light when considering its selective effects on circadian physiology and behaviour. During the application of intermittent light, we did not find any significant dose-dependent effects on acute melatonin suppression, objective alertness or subjective sleepiness. These data run counter to those produced with continuous LE during the biological night, where dose–responses relationships between light intensity and subjective sleepiness [11], electroencephalographic (EEG) theta spectral power density [11] and melatonin suppression [5] are observed. The lack of a dose–response relationship, and the fact that light stimuli only altered subjective sleepiness, together suggest that these findings are underpinned by psychological factors (i.e. being awakened at night to observe flashed light) rather than psychophysical factors (differences in sensations of the light intensities). The distinct subtypes of ipRGCs may also contribute to this, where differences in retinal connectivity, temporal, spatial and intensity signalling, coupled with both distinct and overlapping brain targets [46–52], may lead to divergent light sensitivities depending on the physiologic pathway(s) assayed through the outcome measure(s) selected.

This study is the first to define the intensity sensitivity of the circadian system to sequenced flashes of light presented during the biological night. This paradigm leverages evolutionarily unusual stimuli to drive clinically meaningful shifts in circadian rhythms without substantial changes in acute measures of sleep behaviour and circadian physiology, in contrast to continuous light that often affects such performance markers in undesirable and disruptive ways. The flashed light paradigm is, therefore, a powerful method to drive clinically useful shifts in circadian rhythms, and, further, is orders of magnitude more efficient than continuous light paradigms in terms of time, energy and outcome, which is critical in the development of wearable technology that could be developed as a countermeasure to circadian desynchrony in a variety of environments.

## 4. Material and methods

### (a) Pre-registration and deviations from pre-registered protocol

The study protocol was registered at ClinicalTrials.gov (NCT01119365; ‘Bright Light as a Countermeasure for Circadian Desynchrony’). The variable-duration study was pre-registered on the Open Science Framework (https://osf.io/5sv53/). Notably, we deviated from the pre-registered study protocol by including an additional (10 s) exposure duration. In both the variable-intensity and the variable-duration studies, polysomnography (PSG) data were collected but not analysed.

### (b) Sample characteristics

A total of 59 healthy, young (18–35 years) participants of normal weight with no somatic diseases, no sleep disorders (Pittsburgh Sleep Quality Index, PSQI [53] less than or equal to 5), of moderate chronotype (reduced Morningness-Eveningness Questionnaire, MEQ [54] greater than or equal to 11 and less than or equal to 27), no history of substance abuse (Alcohol Use Disorders Identification Test, AUDIT [55] less than or equal to 7), no depressive symptoms (Center for Epidemiologic Studies Depression scale, CES-D [56] less than or equal to 17), no use of hormonal contraceptives (females only) and normal colour vision (assessed with Ishihara Plates [57]) completed the studies. Females attended the laboratory within 4 days after the onset of menses.

### (c) Variable-intensity study

Twenty-eight participants (14 female and 14 male) completed the study. We excluded one participant from further analyses due to the mistiming of the light stimulus relative to their circadian phase, yielding a total sample of 27 subjects (*n* = 27, mean ± s.d. age: 27 ± 5.16) years. The breakdown of intensity assignment was: 3 lux, four participants; 30 lux, four participants; 95 lux, four participants; 300 lux, three participants; 950 lux, four participants; 3000 lux, four participants; 9500 lux, four participants. All flashes were 2 ms in length.

### (d) Variable-duration study

Thirty-one participants (13 female and 18 male) completed the study. We excluded two participants (1 female and 1 male) because of contaminated melatonin assays, one participant due to mistiming of light (female), and one participant because of accidental LE in the morning (female), yielding a total sample of 27 participants (*n* = 27, mean ± s.d. age: 25.7 ± 3.94 years). The breakdown of duration assignment was: 10 µs, three participants; 100 µs, four participants; 1 ms, four participants; 10 ms, five participants; 100 ms, three participants; 1 s, four participants; 10 s, four participants. All flashes were 2000 lux in intensity.

### (e) Design

Participants were exposed to a sequence of 240 light flashes of varying, logarithmically spaced intensity at fixed duration (2 ms flashes; 3, 30, 95, 300, 950, 3000 or 9500 photopic lux) or varying duration at fixed intensity (10 µs, 100 µs, 1 ms, 10 ms, 100 ms, 1 s, 10 s, 2000 lux) spaced 15 s apart (from onset to onset). Acute effects on melatonin suppression, objective alertness, subjective sleepiness and electrophysiological correlates of arousal (PSG; not reported herein) were measured immediately before and at the end of the LE. Effects of LE on circadian phase was measured as the change in melatonin onset determined on a constant posture protocol (CP1) prior to LE and a constant posture protocol the following day (CP2).

### (f) Protocol

Participants took take part in a 16-day study protocol, as follows.

Days 1–14: participants were instructed to maintain a regular sleep and wake time schedule at home (±30 min window of bedtime and wake time). Sleep–wake patterns were monitored using an actigraph (Actiwatch2, Philips, Bend, OR, USA) and a self-reported sleep diary [58]. From these data, the midpoint of sleep (MSP) was estimated and used as the midpoint of the in-laboratory sleep opportunity.

Day 15: the participant entered the laboratory during the late afternoon of Day 15. During the evening, the participant underwent the first constant posture procedure (CP1, 8 h duration, beginning 8 h before habitual bedtime). During this procedure, the participant was given isocaloric meals (Ensure, Abbott Laboratories, Chicago, IL, USA) every 60 min, yielding a total caloric intake matched to what they would have received during dinner (calculated using the Mifflin-St. Jeor formula; [59]). During CP1, objective alertness (auditory version of the PVT [20,21]) and subjective sleepiness (SSS [22]) were measured every 60 min. Saliva was collected every 30 min in untreated polypropylene tubes. Four hours before MSP (typical bedtime), participants were given the opportunity to sleep in darkness. After 1 h and 45 min of sleep time in darkness (2 h and 15 min before MSP), participants were awakened in the dark (in general, light given up to 2 h after MSP evokes delays in circadian timing). Saliva was collected and auditory PVT and SSS were administered in the dark. Starting 2 h before MSP, participants were exposed to a 60 min sequence of 240 full-field flashes through a custom-made mask for light delivery. At 20, 40 and 60 min into the LE, saliva was collected. During the last 10 min of LE, the auditory PVT was administered, followed by an SSS. The mask was then removed from the participant and the participant continued to sleep in darkness.

Day 16: the participant was awakened at their habitual wake time (4 h after MSP) into a dimly light room (less than 10 lux) and received breakfast and lunch at usual times. During the evening, the participant had a second constant posture procedure (CP2, 10 h in total, beginning 8 h before habitual bedtime), during which the participant was given isocaloric meals every 60 min, yielding a total caloric intake matched to what they would have received during dinner (same number as on Day 15, but spread over 10 h instead of 8). During CP2, objective alertness (auditory PVT) and subjective sleepiness (SSS) were measured every 60 min and saliva was collected every 30 min.

### (g) Stimulus delivery

Binocular full-field flashes of differing durations (2 ms in variable-intensity study; 10 µs, 100 µs, 1 ms, 10 ms, 100 ms, 1 s, 10 s in variable-duration study) were delivered to the participant. A custom-light mask was constructed using modified welding goggles (Jackson WS-80 Shade 5.0 Cutting Goggles; Kimberly-Clark Professional, Mississauga, ON, Canada) containing an acrylic panel with three horizontally arranged LED strips (12 SMD LEDs each, Lumileds L235-4080AHLCBAAC0). The light from the LEDs was diffused using a piece of diffusing acrylic (TAP Plastics, Mountain View, CA, USA). For additional diffusion, the participant wore ping pong ball halves cut out to match the shape of the eye's orbit. The LEDs were pulsed using electronics developed in-house based on the Arduino Uno R3 microcontroller. In the variable-intensity study, we used verified photopic illuminances at 3, 30, 95, 300, 950, 3000 or 9500 photopic lux as confirmed by a calibrated photometer (International Light Technologies ILT900, Peabody, MA, USA).

We verified the timing of our apparatus for the nominal flash durations 10 µs, 100 µs, 1 ms, 10 ms, 100 ms and 1 s using an integrated photodiode and transimpedance amplifier (OPT101, Texas Instruments) connected to a digital oscilloscope (Tektronix TDS 2024C). We measured the logic-level control pulse sent from the microcontroller as well as the light output (electronic supplementary material, figure S2). We averaged over 128 (10 µs), 128 (100 µ*s*), 128 (1 ms), 64 (10 ms), 16 (100 ms) and 16 (1 s) pulses. The maximum amplitude of the pulse is approximately constant across all nominal pulse durations, indicating that there is no shift in light intensity due to duration. Integrating the light output, the logarithm of the integrated light output over the pulse duration is linear with the logarithm of the nominal pulse duration. Spectral output was measured using a PR-670 spectroradiometer (Photo Research, Syracuse, NY, USA) (yielding calibrated radiance) for all stimulus durations, indicating stationarity of the spectrum across stimulus durations and across the entire stimulus protocol (240 flashes, 1 h). The results of these validation measurements are shown in electronic supplementary material, figure S2.

### (h) Spectral and *α*-opic properties of light

The spectrum of the light measured in the corneal plane corresponded to a warmish white light (CIE 1931xy chromaticity: [0.4092, 0.3969]; CCT: 3466 K). The spectrum is visualized in electronic supplementary material, figures S1 and S2, and can be viewed on the luox [60] platform. Metrics related to the recent CIE S026/E:2018 [61] standard are given in table 1 for unit illuminance (1 lux). The spectral invariance with stimulus duration is shown in electronic supplementary material, figure S2. The invariance of the spectrum means the *α*-opic irradiances simply scale proportionally at different illuminances.

**Table 1.** *α*-opic stimulus properties at unit photopic illuminance (1 lux) calculated using the free CIE S 026 *α*-opic Toolbox (v. 1.049, version dated 26 March 2020) implementing the CIE S 026/E:2018 standard [61]. To derive the *α*-opic irradiance and the *α*-opic equivalent daylight (D65) illuminance at other photopic illuminances, multiply the values by the photopic illuminance value. The *α*-opic efficacy of luminous radiation is a scale-invariant ratio that only depends on the relative spectrum.

	S-cone-opic	M-cone-opic	L-cone-opic	Rhodopic	Melanopic
*α*-opic irradiance, (mW·m^–2^)	0.35	1.2	1.6	0.90	0.72
*α*-opic efficacy of luminous radiation, (mW·lm^–2^)	0.35	1.2	1.6	0.90	0.72
*α*-opic equivalent daylight (D65) illuminance (lux)	0.43	0.85	1.0	0.62	0.55

### (i) Melatonin assay

Saliva (at least 1 ml) was collected using polypropylene tubes (Fisher Scientific, Hampton, NH, USA). Samples were centrifuged after collection, frozen at –20°C, then stored at –80°C within 24 h. Salivary melatonin concentration was assayed according to the manufacturer's instructions (Salivary melatonin ELISA no. 3402, Salimetrics, Carlsbad, CA, USA; assay range: 0.78–50 pg ml^−1^, sensitivity = 1.37 pg ml^−1^). For a given participant, all samples were assayed on the same plate.

### (j) Objective alertness: auditory PVT

We used a modified auditory PVT [20,21] to measure objective alertness using a serial collection of simple reaction times to auditory stimuli generated by a piezo buzzer. The stimuli were spaced apart in time with a random ISI between 2 and 6 s (discrete steps: 2, 3, 4, 5, 6 s ISI). Upon button press, the tone stopped and the next trial began with a random ISI. Approximately 100 of these stimuli were presented, with the order of ISI randomized at the beginning of the experiment. This assessment took 10 min. The auditory PVT was implemented using custom-made Arduino hardware and software. To measure response latencies, we modified sample code from a report validating using the Arduino platform to measure reaction times [62]. There is a 30 s time out which is considered a lapse trial. If there is a response during the ISI period, this was counted as an error of commission and the counter was reset, starting a new trial period. The random seed for the ISI is initialized by reading analogue voltage noise from an unconnected pin in the Arduino.

### (k) Subjective alertness: Stanford Sleepiness Scale

Participants completed the SSS [22]. The SSS is a single-question assessment of current sleepiness that uses a 7-point Likert-like scale. Scale values range from 1 to 7, with higher values indicating greater subjective sleepiness.

### (l) Determination of phase shift

Phase shifts were determined by examining the acute change in the timing of salivary DLMO. This onset was determined by calculating the time at which the melatonin concentrations rose above a variable threshold (twice the average of the first three daytime samples [63]). In cases in which this variable threshold was ambiguous due to a noisy baseline (*n* = 2 variable-intensity, *n* = 2 variable-duration), we manually set the threshold to 10 pg ml^−1^. Determination of ambiguity was made blind to the lighting parameters to which the participant was exposed. Phase shift was calculated as the DLMO on CP1—DLMO on CP2, such that negative changes indicate a delay in timing.

### (m) Statistical analysis

Dose–response curve fitting was done in OriginPro 2021 (OriginLab, Northampton, MA, USA). Outlier detection was done with Grubbs's test (https://www.graphpad.com/quickcalcs/grubbs1/). Data to anchor the non-specific effects of the protocol on outcome measures were previously published [41]. For data failing to achieve a dose–response, data were analysed using simple intercept + slope linear models using the lm() function in R.

## Data Availability

The data that support the findings of this study are available from the Dryad Digital Repository: https://doi.org/10.5061/dryad.4f4qrfjcv [64].
